# Supplementary material for: Patterns of Intron Gain and Loss in Fungi
Source: PLoS Biol. 2004 Nov 30;2(12):e422. doi: 10.1371/journal.pbio.0020422 (PMC532390; doi:10.1371/journal.pbio.0020422)
Supplement: Table S1 — Also available at http://genes.mit.edu/NielsenEtAl/. (4.3 MB ZIP). [file pbio.0020422.st001.zip › NielsenEtAl/html/1284.html]

AN0990.1.NCU03479.1.MG01804.1.FG00819.1


```
 CLUSTAL W (1.82) Multiple Sequence Alignments - Introns Inserted


Sequence 1: MG01804.1	829 aa
Sequence 2: FG00819.1	864 aa
Sequence 3: NCU03479.1	850 aa
Sequence 4: AN0990.1	884 aa
Alignment Length: 944 aa
Number Identitical Residues: 494 aa
Alignment Score (without introns) 22254


MG01804.1 	MASKRKASAMNVAAPDEPVDPADELVFTCLGGGNEVGRSCHIIQYKGKTVM0LDAGQHPA
NCU03479.1	MASKRKASAM-ATEPEEPVDPADELMFLNLGGGNEVGRSCHIIQYKGKTVM0LDAGQHPA
FG00819.1 	MASKRKAAAMNAAAAEEPVDPSDELMFLCLGGGNEVGRSCHIIQYKGKTVM0LDAGQHPA
AN0990.1  	MAAKRKAAAMNAVDD-EPVDPSDELAFYCLGGGNEVGRSCHIIQYKGKTVM0LDAGMHPA
          	**:****:**...   *****:*** *  ********************** **** ***

MG01804.1 	YDGLAALPFFDDFDLSTVDVLLISH2--------------------------~-----FH
NCU03479.1	YDGLAALPFFDDFDLSTVDVLLISH2--------------------------~-----FH
FG00819.1 	YDGLAALPFYDDFDLSTVDVLLISH2PVQDTTALYCHGQYCACVMSISMIML0LIGHSFH
AN0990.1  	KEGFSALPFFDEFDLSTVDILLISH2--------------------------~-----FH
          	 :*::****:*:*******:*****                                 **

MG01804.1 	VDHAASLPYVLSKTNFKGRVFMTHPTKAIYKWLIQDSVRVGNTSSNP-TSQPVYTEQDHL
NCU03479.1	IDHAASLPYVLAKTNFRGRVFMTHATKAIYKWLIQDSVRVGNTSSNP-QSSLVYTEEDHL
FG00819.1 	IDHAASLPYVLAKTNFRGRVFMTHPTKAIYKWLIQDSVRVGNTSSNP-TTQPVYTEQDHL
AN0990.1  	VDHSSALPYVLSKTNFKGRVFMTHATKAIYKWLIQDNVRVNNTASSSDQRTTLYTEHDHL
          	:**:::*****:****:*******.***********.***.**:*...    :***.***

MG01804.1 	NTFPQIEAIDYYTTHTISSIRITPYPAGHVLGAAMFLIEIAGMNIFFTGDYSREQDRHLV
NCU03479.1	KTFPMIEAIDYNTTHTISSIRITPYPAGHVLGAAMFLIEIAGLKIFFTGDYSREEDRHLI
FG00819.1 	NTFPQIEAIDYHTTHTISSIRITPYPAGHVLGAAMFLIEIAGLNIFFTGDYSREQDRHLV
AN0990.1  	STLPLIETIDFNTTHTINSIRITPYPAGHVLGAAMFLISIAGLNILFTGDYSREEDRHLI
          	.*:* **:**: *****.********************.***::*:********:****:

MG01804.1 	SAEVPRGVKIDVLITESTYGIASHVPRVEREQALMKSITGILNRGGRVLMPVFALGRAQE
NCU03479.1	SAKVPKGVKIDVLITESTYGIASHIPRPEREQALMKSITGILNRGGRVLMPVFALGRAQE
FG00819.1 	SAEVPKGVKIDVLITESTYGIASHVPRLEREQALMKSITSILNRGGRVLMPVFALGRAQE
AN0990.1  	PATVPRGVKIDVLITESTFGISSNPPRLEREAALMKSITGVLNRGGRVLMPVFALGRAQE
          	.* **:************:**:*: ** *** *******.:*******************

MG01804.1 	LLLILDEYWGKHQEYQKVPIYYASNLARKCMVVYQTYVGAMNDNIKRLFRERLAEAEASG
NCU03479.1	LLLILDEYWGKHAEYQKYPIYYASNLARKCMLVYQTYVGSMNDNIKRLFRERLAESESSG
FG00819.1 	LLLILDEYWGKHADFQKYPIYYASNLARKCMLIYQTYVGAMNDNIKRLFRERMAEAEASG
AN0990.1  	LLLILEEYWETHPELQKIPIYYIGNTARRCMVVYQTYIGAMNDNIKRLFRQRMAEAEASG
          	*****:*** .* : ** **** .* **:**::****:*:**********:*:**:*:**

MG01804.1 	KSGAGGGGPWDFKYIRSLKNLDRFDDLGPCVMLASPGMLQNGVSRELLERWAPSDKNGVV
NCU03479.1	-DGAGKGGPWDFRFIRSLKSLDRFEDVGGCVMLASPGMLQNGVSRELLERWAPSEKNGVI
FG00819.1 	-DGAGKGGPWDFKYIRSLKNLDRFDDVGGCVMLASPGMLQNGVSRELLERWAPSEKNGVI
AN0990.1  	-DKSVSAGPWDFKYVRSLRSLERFDDVGGCVMLASPGMLQTGTSRELLERWAPNERNGVV
          	 . :  .*****:::***:.*:**:*:* ***********.*.**********.::***:

MG01804.1 	ITGYSVEGTMAKQIMQEPEQIPAIMTRNAAAASRR-----ADGEKPMIPRRCSVAEFSFA
NCU03479.1	ITGYSVEGTMAKQLLQEPEQIQAVMSRNIAGARRGPG---GDAEKVMIPRRCTVQEFSFA
FG00819.1 	ITGYSVEGTMAKQIMQEPDQIQAVMSRSMAGARRMPG---GDGEKVLIPRRCSVQEYSFA
AN0990.1  	MTGYSVEGTMAKQLLNEPDQIHAVMSRAATGMGRTRMNGNDEEQKIMIPRRCTVDEISFA
          	:************:::**:** *:*:*  :.  *   ... : :* :*****:* * ***

MG01804.1 	AHVDGTENREFIEEVAAPVV0ILVHGEQNNMMRLKSKLLSLNATKTEKVKIYSPRNCEEL
NCU03479.1	AHVDGVENREFIEEVAAPVV~ILVHGEVHNMMRLKSKLLSLNATKEHKVKVFSPRNCEEL
FG00819.1 	AHVDGVENREFIEEVQAPVV0ILVHGEQHNMMRLKSKLLSLNANKTAKVKVYSPRNCEEL
AN0990.1  	AHVDGVENRNFIEEVSAPVV0ILVHGEKHQMMRLKSKLLSLNAEKTVKVKVYTPANCEEV
          	*****.***:***** **** ****** ::************* *  ***:::* ****:

MG01804.1 	RIPFKTDKTAKVVGKLASITPPTRLPTDDDY-----HLHPQLVTGVLVQNDFKLSLMAPE
NCU03479.1	RIPFKTDKVAKVVGKLASIPPSLKEAKTGHDGPLPSSTEPQLITGVLVQNDFKMSLMAPE
FG00819.1 	RIPFKADKIAKVVGKLACIQPPQ--SIHPDQ-----TATPPLVTGVLVQNDFKLSLMAPE
AN0990.1  	RIPFRKDKIAKVVGKLAQTTLPTD-NEDGDG---------PLMAGVLVQNGFDLSLMAPD
          	****: ** ********    .       .           *::******.*.:*****:

MG01804.1 	DLREYAGLTTTTIVCRQKIMLAAAGVDLIRWALESTFGAVDELP---EMR-QLHKEVP-N
NCU03479.1	DLREYAGLTTTTIACKQRLKLSAAGIDLIKWGLEGTFGAVEELP---EVKPKLEIVKSEN
FG00819.1 	DLREYAGLNTTTITCKQRLTLSAAGVDLVKWALEGTFGNIEELP---EMR-RAKNGQNGH
AN0990.1  	DLREYAGLATTTITCKQHITLSSASMDLIKWALEGTFGAIEEIGTDEDAEKEDQQSESEE
          	******** ****.*:*:: *::*.:**::*.**.*** ::*: :..: . . .     .

MG01804.1 	GDDMILDAKEE---EVVDEEVERPVAGYIVMGSINVRCFG-SGEVELEWEGNTMNDGFAD
NCU03479.1	GDTKMEEADEELP------HGDDVVAAYLVMGCVTVRYRA-SGEVELEWEGNMLNDGIAD
FG00819.1 	ADKMITDGDDESKQEDADEEVASLVAAYLVMGCVSVRYRT-NGEVELEWEGNMLNDGIAD
AN0990.1  	KQRMKEEADEEIP--------MEKPQAYLVMGCVVIRYHPRTREVELQWEGNMMNDGIAD
          	 :    :..:*               .*:***.: :*    . ****:**** :***:**

MG01804.1 	AVMAVLLTVESSPAAVKK1SA--SKHSHSHAPQEEYVLPTRNLHARLSPEERLERLFMFL
NCU03479.1	AVMAVLLGIESSPAAVKR1SA--TKNPHTHSP----LPADKNPHSHLTPEDRFFRLCMFL
FG00819.1 	SVMAVLFSVESSPAAVKR~SS--AKHSHSHD------LPEVNPHHSATPEERLERLLWFL
AN0990.1  	AVMAVLLTVESSPASVKQ1SAKHNKHHHHHHHDETDTLKFLNPHAAQDAEERFARLLMML
          	:*****: :*****:**: *:.  *: * *  ..       * *    .*:*: **  :*

MG01804.1 	EAQFGADHVSPIKDPRLP------------------VSDDTSGEAENGD----DDGGRQQ
NCU03479.1	EAQFGQDNVSPIVEPKLPPLSPTTKAITSPSEETAKSSDVKSDADADASMDVSEEDEDEQ
FG00819.1 	EAQFGQDNVAPVTTPKLPPLE--------------DVDDKNKNEGEDEDAMKTDEDGEDA
AN0990.1  	EAQFGSD-IAPIERPRVPSSTES-------ATTTTNGNGNSKSDSEQLSSLESKTDGATP
          	***** * ::*:  *::*.   :       :  ::. .. ...   : .   :. .    

MG01804.1 	R----LEERQRAEIERLHKIGIPVPGVAIKIDDKLNAKVWLEDLEVECANKAFGDRVRVV
NCU03479.1	Q----LKARERAEVERLERMGIPKPGVRIKVD-KMEAKVWLEDLEVECANKIFRERVRAV
FG00819.1 	Q----LQERQQKEIERLHKIGIPVPGVSIKVD-KMSATVWLEDLEVESSHKVFADRVRAV
AN0990.1  	QDPDTLSELEAAELSRLHALGIPVPGIEIKVD-KHVARVWLEDLEVECANAVLRDRVRVV
          	:...:*.  :  *:.**. :*** **: **:* *  * *********.::  : :***.*

MG01804.1 	VERAVEVTAPLWG------------------------------------
NCU03479.1	VERAVEVVAPLWG------------------------------------
FG00819.1 	VERAIEVTAPLWG------------------------------------
AN0990.1  	IERAVETVASMWSVGRSSKTITNGGGKEIAGTGADDVASKPGLEVAARA
          	:***:*..*.:*. . ::.: :...... :.:.:.. ::... . :: :
```
